# SENP1 regulates the transformation of lung resident mesenchymal stem cells and is associated with idiopathic pulmonary fibrosis progression

**DOI:** 10.1186/s12964-022-00921-4

**Published:** 2022-07-14

**Authors:** Wei Sun, Xiaoshu Liu, Xiaoyu Yang, Xiaoyan Jing, Chunyan Duan, Ganghao Yang, Chi Wu, Hui Huang, Qun Luo, Shu Xia, Qian Zhang, Yang Yang, Zuojun Xu

**Affiliations:** 1 Department of Respiratory and Critical Medicine, Sichuan Provincial People’s Hospital, Sichuan Academy of Medical Sciences, No. 32, Section 2, West 1st ring road, Qingyang District, Chengdu, 610072 Sichuan China; 2 Department of Respiratory and Critical Medicine, Peking Union Medical College Hospital, Chinese Academy of Medical Sciences and Peking Union Medical College, No. 1 Shuai Fu Yuan Street, Dong Cheng District, Beijing, 100730 China; 3 State Key Laboratory of Respiratory Disease, National Clinical Center for Respiratory Disease, Guangzhou Institute of Respiratory Health, The First Affiliated Hospital of Guangzhou Medical University, Guangzhou, Guangdong China

**Keywords:** Idiopathic pulmonary fibrosis, Myofibroblasts, Lung resident mesenchymal stem cells, SENP1

## Abstract

**Background:**

Lung resident mesenchymal stem cells (LR-MSCs) play an important role in idiopathic pulmonary fibrosis (IPF) by transforming into myofibroblasts, thereby losing their repair ability. Evidence suggests that key proteins of multiple signaling pathways are involved in myofibroblast differentiation of LR-MSCs, such as β-Catenin and GLI family zinc finger 1 (GLI1). These proteins are regulated by SUMO (small ubiquitin-like modifier) modification, which is a post-translational modification that promotes protein degradation, while Sumo specific protein 1 (SENP1)-mediated deSUMOylation produces the opposite biological effects. Therefore, we speculated that SENP1 might be a potential target for treating pulmonary fibrosis by preventing the myofibroblast differentiation of LR-MSCs.

**Methods:**

LR-MSCs were isolated from mice by using immunomagnetic beads. The extracted LR-MSCs were identified by flow cytometric analysis and multilineage differentiation assays. Lentivirus packaged shRNA silenced the expression of SENP1 in vitro and vivo. The silencing efficacy of SENP1 was verified by real-time quantitative PCR. The effect of down-regulated SENP1 on the myofibroblast differentiation of LR-MSCs was assessed by Immunofluorescence and Western blot. Immunoprecipitation was used to clarify that SENP1 was a key target for regulating the activity of multiple signaling pathways in the direction of LR-MSCs differentiation. LR-MSCs resident in the lung was analyzed with in vivo imaging system. HE and Masson staining was used to evaluate the therapeutic effect of LR-MSCs with SENP1 down-regulation on the lung of BLM mice.

**Results:**

In this study, we found that the myofibroblast differentiation of LR-MSCs in IPF lung tissue was accompanied by enhanced SENP1-mediated deSUMOylation. The expression of SENP1 increased in LR-MSCs transition of bleomycin (BLM)-induced lung fibrosis. Interfering with expression of SENP1 inhibited the transformation of LR-MSCs into myofibroblasts in vitro and in vivo and restored their therapeutic effect in BLM lung fibrosis. In addition, activation of the WNT/β-Catenin and Hedgehog/GLI signaling pathways depends on SENP1-mediated deSUMOylation.

**Conclusions:**

SENP1 might be a potential target to restore the repair function of LR-MSCs and treat pulmonary fibrosis.

**Video Abstract**

**Supplementary Information:**

The online version contains supplementary material available at 10.1186/s12964-022-00921-4.

## Background

Idiopathic pulmonary fibrosis (IPF) is an interstitial lung disease with unknown etiology and chronic progression [1]. The median survival time is less than 3 years [2]. Studies have shown that the proliferation of myofibroblasts induced by chronic injury in alveolar epithelial cells (AECs) is the core event leading to the development of IPF [3, 4]. Therefore, the key to IPF treatment is protecting or maintaining the repair and regeneration ability of AECs and inhibiting the accumulation of myofibroblasts.

Recent studies have found that mesenchymal stem cells (MSCs) with multilineage differentiation potential play an essential role in self-renewal ability [5]. MSCs have been found to reside in interstitial perivascular niches in the lung, these lung-resident mesenchymal stem cells (LR-MSCs) regulating the proliferation of myofibroblasts and possessing typical characteristics shared by other tissue-specific MSCs, including self-renewal ability [6–8]. If LR-MSCs differentiate into myofibroblasts, they will be unable to repair damaged lung tissue and secrete extracellular matrix components, such as collagen fibers, to accelerate pulmonary fibrosis [9, 10]. Therefore, the transformation of LR-MSCs into myofibroblasts might underlie the progression of IPF. However, the specific mechanism regulating the transformation of LR-MSCs remains unknown. Studies have shown that the transformation of LR-MSCs involves multiple signaling pathways, such as WNT/β-catenin, Hedgehog/GLI family zinc finger (GLI), and the over-activation of these signaling pathways maintains the transformation of LR-MSCs into myofibroblasts [11, 12]. Predictably, simultaneous regulation of critical proteins of these signaling pathways could effectively inhibit the transdifferentiation of LR-MSCs would represent a new strategy to treat IPF.

Small ubiquitin-like modifier (SUMOylation) is an essential post-translational modification of proteins, promoting protein degradation via ubiquitination by binding to lysine residues [13]. SUMOylation of proteins exists in a state of dynamic equilibrium within the cytoplasm. SUMO specific protein 1 (SENP1)-mediated deSUMOylation resulting in protein accumulation by shearing the SUMO chain on lysine residues [14]. The level of key effector proteins of the WNT/β-Catenin and Hedgehog/GLI signaling pathways (such as β-Catenin and GLI1) was modified by deSUMOylation. SENP1 acts as endopeptidase that can maintain the stabilizations and activities of β-Catenin and GLI1 via deSUMOylation [15, 16]. Therefore, we speculated that SENP1-mediated deSUMOylation might be an important mechanism that regulates the transformation of LR-MSCs, and intervention with this process might restore the repair function of LR-MSCs and inhibit the progress of IPF.

This study found that transdifferentiation of LR-MSCs into myofibroblasts exists in the lung tissue of patients with IPF. Overexpression of SENP1 was also found in myofibroblastic differentiation of LR-MSCs. Downregulation of SENP1 in LR-MSCs could reverse the transformation of LR-MSCs into myofibroblasts and restore their repair function, thus ameliorating pulmonary fibrosis of the mice BLM model. The mechanism by which SENP1-mediated deSUMOylation affects the transformation of LR-MSCs involves promoting the SUMOylation of key proteins in the WNT/β-Catenin and Hedgehog/GLI signaling pathways, consequently enhancing the degradation of β-Catenin and GLI1.

## Methods

### Collection of samples from patients with IPF

Samples of lung tissues from 6 patients with IPF were obtained from the Department of Lung Transplantation, The First Affiliated Hospital of Guangzhou Medical University, Guangzhou, China. All diagnoses of IPF were made following the American Thoracic Society (ATS) and European Respiratory Society (ERS) criteria for IPF 2011. Normal peripheral tissues from patients with tumors (n = 6) were used as controls and were supplied by the Thoracic Surgery Department of Peking Union Medical College Hospital, Chinese Academy of Medical Sciences. All patients were male (Additional file 1: Table S1). Informed consent was obtained from all the patients. The Ethics Committee approved this study of Peking Union Medical College Hospital (JS-1127).

### Animal care and mouse pulmonary fibrosis models

Male C57BL/6 mice (Six‐to eight‐weeks‐old, specific pathogen-free (SPF), Animal Center, Peking Union Medical College Hospital, Chinese Academy of Medical Sciences, Beijing, China) were obtained from the Laboratory Animal Center of Chinese Academy of Medical Sciences and housed at a constant room temperature with a 12 h light/dark cycle. Standard rodent chow and water were provided ad libitum. The animal experiments were conducted according to the regulations established by the Institutional Committee for the Care and Use of Laboratory Animals and were approved by the Chinese Academy of Medical Sciences Laboratory Animal Center; all efforts were made to minimize their suffering. A single surgeon conducted all surgical procedures under aseptic conditions in the Laboratory Animal Unit. Mice were anesthetized with isoflurane inhalation. The mice were injected intratracheally with 50 µL of 5 mg/kg BLM. On day 14, after bleomycin treatment, the mice were sacrificed, and their lungs were collected for subsequent experiments.

### LR-MSC isolation and LR-MSC-myofibroblast transition model in vitro

Lung single-cell suspensions were prepared from the lungs of C57BL/6 mice (4–6 weeks old). The lung was diced and incubated with an enzyme mixture containing 0.2% collagenase I (Sigma, St. Louis, MO, USA), 2.4 U/mL dispase (Sigma), and 0.001% DNAse (Sigma) for 1 h at 37 °C with shaking. After centrifugation, the lung cells were resuspended in Hank’s buffered salt solution, and then filtered through 100 μm and 40 μm filters. Mouse LR-MSCs were purified by staining for stem cell antigen-1 (Sca-1), protein tyrosine phosphatase receptor type C (CD45), and platelet and endothelial cell adhesion molecule 1 (CD31) using fluorescence-activated cell sorting (FACS) (FACSAria II, BD Biosciences, San Jose, CA, USA) or Magnetic-activated cell sorting (MACS) (Miltenyi Biotec, Bergisch Gladbach, Germany). Freshly isolated LR-MSCs were cultured at a concentration higher than 10^5^ cells/mL with DMEM containing 10% fetal bovine serum, 4% L-glutamine, 1% nonessential amino acids, and 1% penicillin and streptomycin and maintained in a humidified atmosphere of 95% air and 5% CO_2_ at 37 °C. The medium was changed every three days, and cells were passaged 1:2 using 0.25% trypsin when they reached 70–90% confluence. The primary LR-MSCs were passaged for two generations for transdifferentiation in vitro. Recombinant TGFβ1 (Proteintech Group, Wuhan, China) was used at a 10 ng/mL concentration to induce LR-MSC myofibroblast transition.

### Flow cytometry analysis

Mouse LR-MSCs were incubated with fluorescently labeled antibodies at 4 °C for 20 min in the dark, followed by two washes with phosphate-buffered saline (PBS). Flow cytometry was performed on a FACSCalibur flow cytometer (Becton Dickinson, Franklin Lakes, NJ, USA), and the data were analyzed using FlowJo software (Tree Star, Ashland, OR, USA). The antibodies used included fluorescein isothiocyanate (FITC)-conjugated anti-Sca-1(Miltenyi Biotec), anti-endoglin (CD105) (Abcam, ab184667), anti-CD90 (Proteintech); phycoerythrin (PE)-conjugated anti-CD31 (BD Pharmingen), anti-CD34 (BD Pharmingen), and anti-CD45 (BD Pharmingen).

### Multilineage differentiation assays

Mouse LR-MSCs were seeded at a density of 4 × 10^3^ cells/cm^2^ in 12-well plates in a standard growth medium and grown to 70% confluence. Their osteogenic differentiation potential was evaluated by culturing cells for 3 weeks in an osteogenic induction medium, comprising a basic medium supplemented with 100 nM dexamethasone and 50 μg/mL ascorbic acid and 10 mM β-glycerophosphate. Then, the deposited calcium phosphate mineral phase was subjected to alizarin red staining. The adipogenic differentiation capacity was confirmed by culturing LR-MSCs in adipogenic induction medium, comprising basic medium with 5 μg/mL insulin, 0.1 μM dexamethasone, 50 μM indomethacin, and 500 μM isobutylmethylxanthine. After 3 weeks, lipid droplets were stained with oil red O solution. For chondrogenesis differentiation, confluent cells were cultured in MSC culture growth media supplemented with 75 Mm of ascorbate, 0.1 mM of dexamethasone, 1 mM of sodium pyruvate, 0.75 mM of proline and 50 ng/ML of TGF-β3. Medium was changed twice per week for 3 weeks. Cells were fixed with 10% formalin for 20 min and stained with Alcian Blue.

### Immunofluorescence analysis of lung tissue from human IPF patient

We followed the methods of Sun et al. [17]. Four percent paraformaldehyde‐fixed, paraffin‐embedded blocks of lung tissues were cut into 4 μm thick sections. The sections were placed on polylysine-coated slides and incubated in a 60 °C oven. The slides were dewaxed using xylene and rehydrated using a gradient of alcohol concentrations. The slides were then placed in a microwave oven until the antigen retrieval solution reached 100 °C for 10 min one and for 2 min four times, cooled to room temperature for 20 min and washed with PBS for 5 min. Primary antibodies against CD90 (Cell Signaling, #13,801, 1:200; Abcam, Ab181469, 1:200), SENP1 (Abcam, Ab236094, 1:200), and alpha-smooth muscle actin (α-SMA) (Abcam, Ab240654, 1:200) were incubated with the slides overnight at 4 °C. Secondary antibodies were incubated with the samples for 1 h at room temperature. Then, the sections were mounted using Fluorescent Mounting Media containing 4′,6-diamidino-2-phenylindole (DAPI) (Abcam). Each tissue section was observed under a confocal laser scanning microscope (Leica SP8, Wetzlar, Germany) at magnifications of 200× and 400×, if necessary.

### Immunofluorescence staining in LR-MSCs

LR-MSCs were fixed with freshly prepared 4% paraformaldehyde for 10 min at room temperature. The cells were then washed three times with PBS. Cells under coverslips were then incubated in 1% bovine serum albumin (BSA). Primary antibodies against α-SMA (Abcam, Ab240654, 1:200), Collagen I (Abcam, Ab260043, 1:200; Abcam, Ab88147, 1:200), and SENP1 (Abcam, Ab236094, 1:200) were incubated with the cells overnight at 4 °C. After washing with PBS, secondary antibodies were incubated with the cells for 1 h at room temperature in a darkened humidified chamber. Finally, cells under coverslips were washed with PBS and mounted in fluorescent mounting medium with DAPI. Images were acquired using a Leica SP8 confocal laser scanning microscope at magnifications of × 200.

### Immunofluorescence staining of mouse lung tissues

We followed the methods of Sun et al. [17]. Four percent paraformaldehyde-fixed mouse lung tissues were cut into 4 μm thick sections. After dehydration, the sections were collected on Superfrost Plus glass slides. Sections were rinsed with PBS and permeabilized with a 1% Triton solution for 5 min. The cells were then blocked with 1% BSA for 1 h. Primary antibodies against α-SMA (Abcam, Ab240654, 1:200), SENP1 (Abcam, Ab236094, 1:200), and Sca-1 (Abcam, Ab51317, 1:200) were incubated with the sections overnight at 4 °C. Secondary antibodies were incubated with the samples for 1 h at room temperature. The sections were mounted with Fluorescent Mounting Media with DAPI and observed under a Leica SP8 confocal laser scanning microscope at magnifications of × 200.

### Inhibition of SENP1 expression

Chemically synthesized *SENP1* shRNAs were designed to target the SENP1 gene (GenePharma, Shanghai, China). The shRNA sequences were identified using the mouse genome database to assess possible crossreactivity. The mouse sequences of the shRNAs were as follows: SENP1-1 (5′-GCAGGATCCTCTTGCAATACC-3′), SENP1-2 (5′-GCACCTCATCAGCCAAATAGC-3′), SENP1-3 (5′-GCATTCCGCTTGACCATTACA-3′). Lentiviral particles were generated based on a standardized protocol using highly purified plasmids and EndoFectinLenti and TiterBoost reagents. LR-MSCs were transfected with *SENP1* shRNAs infected with lentiviral vectors and incubated for 48 h in vitro. *SENP1* shRNAs carried by lentiviral were intratracheally injected to silent the SENP1 expression in lung tissues of mice. Real time-PCR confirmed downregulation expression in vitro and in vivo.

### Labeling and tracing of LR-MSCs

Scramble-shRNAs and LV-*SENP1*shRNAs were cloned into the multi cloning site of the lenti-SENP1-CMV shuttle vector containing a GFP reporter gene. GFP^+^ LR-MSCs were cultured in a “complete expansion medium” (CEM). CEM was supplemented with 1 μg/mL Puromycine (Invivogen) to induce GFP expression. Cultures were harvested twice a week using trypsin–EDTA (Invitrogen) and passaged at a 1:3 ratio in 15 ml CEM in T75 culture flasks. Cell preparations were kept on ice until cell infusion. GFP^+^ LR-MSCs resident in the lung was analyzed with in vivo imaging system (IVIS Lumina) in all treated mice.

### Western blotting analysis

Total protein was collected according to the methods described in our previous report. The protein concentration was assessed using a Thermo Fisher bicinchoninic acid (BCA) kit (Thermo Fisher Scientific, Waltham, MA, USA). Protein samples were separated using 10% SDS-PAGE and transferred to polyvinylidene fluoride (PVDF) membranes. The membranes were incubated with primary antibodies diluted to the recommended concentration, including mouse anti-α-SMA (Abcam, Ab240654, 1:500), rabbit anti-collagen I (Abcam, Ab260043 1:500), rabbit anti-SENP1 (Abcam, Ab236094, 1:500), rabbit anti-β-Catenin (Abcam, Ab32572, 1:500), and rabbit anti-GLI1 (Abcam, Ab217326, 1:500), rabbit anti-SPC (Abcam, Ab211326, 1:500) overnight at 4 °C. The membranes samples were incubated with horseradish peroxidase (HRP)-conjugated secondary antibody at room temperature for 1.5 h, and the protein bands were detected using a chemiluminescence device.

### Immunoprecipitation

LR-MSCs from different groups were collected, washed twice with ice-cold PBS, dissolved in cold radioimmunoprecipitation assay (RIPA) lysis buffer, and then incubated on ice for 30 min. After centrifugation at 12,000 × g for 15 min at 4 °C, Cell lysates (500 μg) were then incubated with 2 μg of anti-β-catenin or anti-GLI1 antibody for 2 h at 4 °C on a rocker platform (30 rocks/min). Protein-antibody complexes were collected with 20 μl of Protein G PLUS Agarose (Santa Cruz Biotechnology, CA, USA) on a rocker platform (30 rocks/min) overnight at 4 °C. The immunoprecipitates were washed three times with lysis buffer. Equal amounts (10 μg/lane) of proteins were subjected to 12% SDS-PAGE for SUMO1 antibodies (1:50; Cell Signaling Technology, Danvers, MA, USA) at 4 °C.

### RT-PCR

Total RNA was extracted from the cells and lung tissues using TRIzol according to the manufacturer’s instructions (Transgene, Beijing, China). The total RNA was subjected to reverse transcription (RT) reaction using a SYBR PrimerScript RT-PCR Kit (Takara, Dalian, China) according to the manufacturer’s instructions. The resultant cDNA was used as the template for the quantitative real-time PCR step using a LightCycler instrument (Roche, Mannheim, Germany) according to the manufacturer’s manual. The relative quantification of target gene expression was performed using glyceraldehyde-3-phosphate dehydrogenase (GAPDH) mRNA as the internal control. The targeted genes were amplified using the following primers: 5′-CTACAAGAAGCCCAGCCTATCGTC-3′ (forward) and 5′-GTCACCTGAGCCAAGGAAACTG-3′ (reverse).

for *SENP1*; 5′-CCTAGCTGGACTGCAGAA-3′ (forward) and 5′-CACCACTGGCCAGAATGATGA-3′ (reverse) for β-Catenin; and 5′-CTGCAGCTGCAGACGGTTATC-3′ (forward) and 5′-AGCCTCCTGGAGATGTGCAT-3′ (reverse) for GLI1;5′- GGTGGTCTCCTCTGACTTCAACA-3′ (forward) and 5′- ACCAGGAAATGAGCTTGACAAAG-3 ′(reverse) for GAPDH.

### Histological analysis of lung tissues

Patients with IPF or mouse specimens were fixed in 10% formaldehyde for 24 h. After dehydration, they were embedded in paraffin, and 1.5 μm thick cross-sections were stained with hematoxylin and eosin (HE), Masson’s trichrome. The sections were evaluated from five randomly selected fields by an independent pathologist (magnification of × 100). The extent of fibrosis was scored as 0 (negative), 1 (weak), 2 (medium), or 3 (intense). Each tissue section was observed under a light microscope (Olympus IX71, Tokyo, Japan) at magnifications of × 200.

### Statistical analysis

Results are presented as mean values ± standard deviations. Statistical significance between two groups was estimated using the unpaired two-tailed Student’s *t*-test. Multiple comparisons of parametric data were performed using a one-way analysis of variance (ANOVA). Nonparametric data were compared with the Mann–Whitney U-test to identify differences between groups. A value of *P* < 0.05 was considered to indicate statistical significance. All statistical analyses were performed using the statistical package SPSS (version 21.0, IBM, Inc., USA).

## Results

### LR-MSCs of IPF tend to differentiate into myofibroblasts

**Fig. 1:**
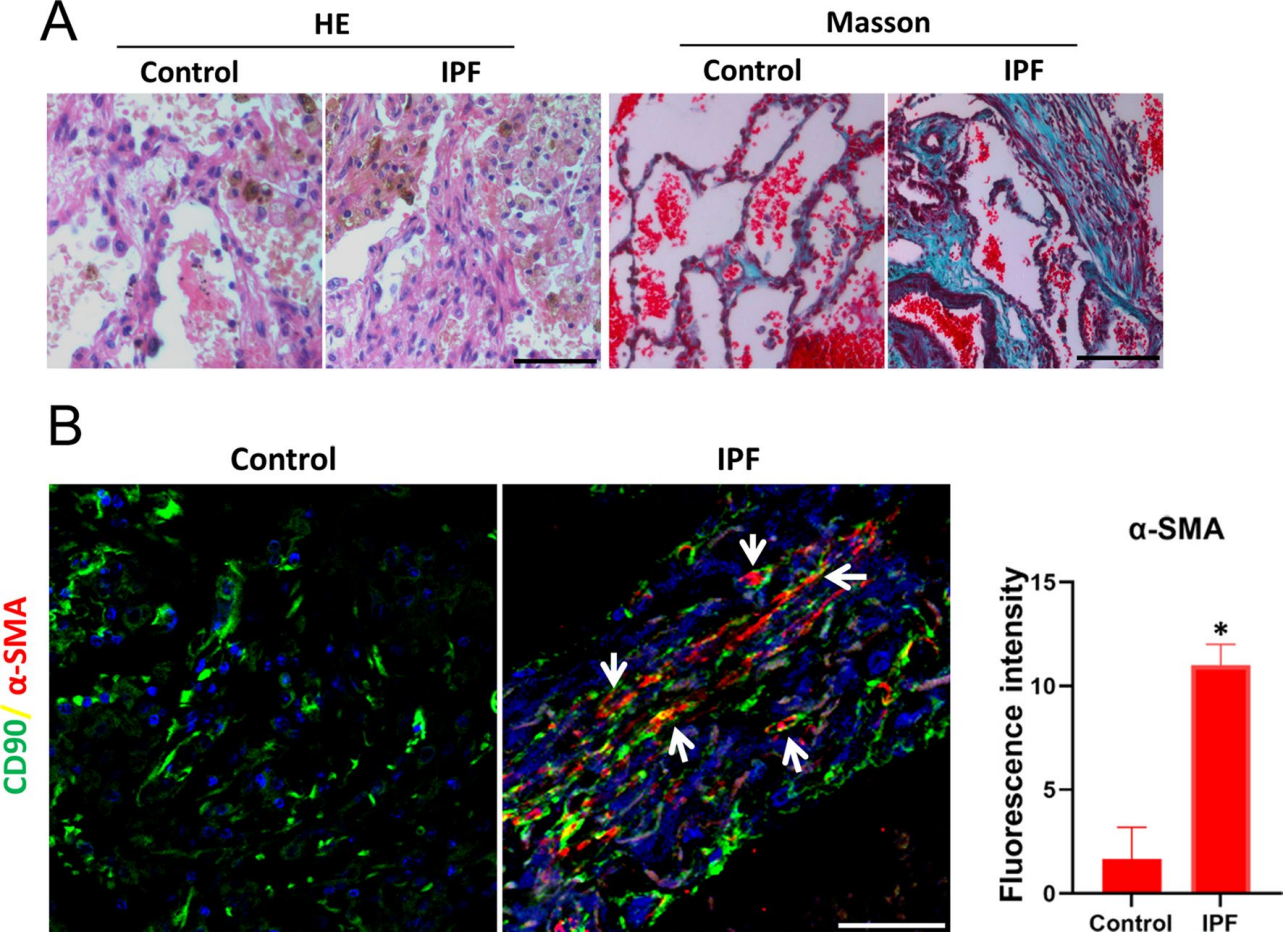
LR-MSCs-myofibroblast transdifferentiation in patients with IPF. **A** Representative results of H&E staining and Masson staining in normal lung and lungs from patients with IPF. **B** Representative images of dual staining for CD90 (green) and α-SMA (red). White arrow indicates LR-MSCs. Quantification is shown in the right panel. *P < 0.05, IPF group *vs.* control group. Scale bar, 50 μm

Lung tissue sections from patients with IPF showed destruction of the lung structure, characterized by accumulation of myofibroblasts and Collagen in the interstitium, as indicated by hematoxylin and eosin (H&E), Masson staining (Fig. 1A). Interestingly, immunofluorescence staining showed that CD90 labeled myofibroblasts (expressing α-SMA) appeared in the lung interstitium (Fig. 1B). These data indicated that LR-MSCs act as a source for myofibroblasts and participate in the progression of IPF.

### SENP1-mediated deSUMOylation of LR-MSCs is increased in IPF

**Fig. 2:**
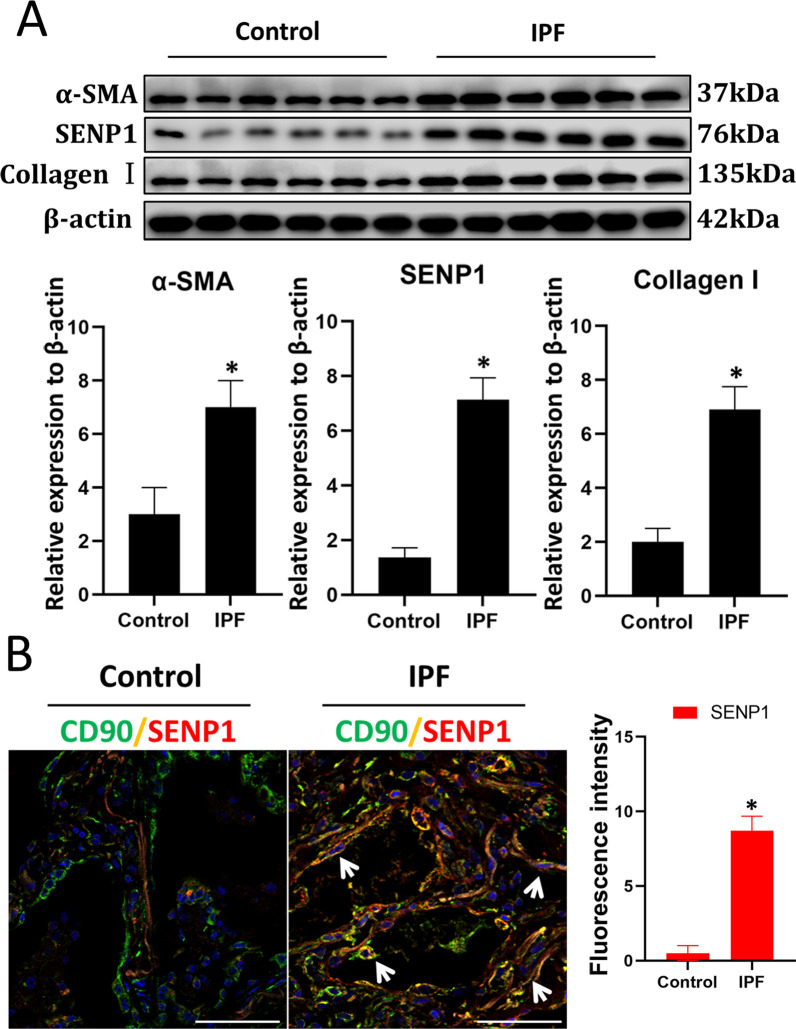
SENP1 mediated deSUMOylation increased in LR-MSCs-myofibroblast of lung tissue from patients with IPF. **A** Western blotting was applied to detect the levels of SENP1, α-SMA, and Collagen I. Quantification is shown in the lower panel.*P < 0.05, IPF group *vs*. control group. **B** Representative images of dual staining for CD90 (green) and SENP1 (red). The white arrow indicates LR-MSCs. Quantification is shown in the right panel. *P < 0.05, IPF group *vs.* control group. Scale bar, 50 μm. The results are shown as the means ± SD

Western blotting showed that in IPF tissue, the level of SENP1, α-SMA, and Collagen I were significantly higher than that in normal tissue (Fig. 2A). To evaluate the role of deSUMOylation in myofibroblast differentiation of LR-MSCs, we performed immunofluorescence double staining of SENP1 with CD90 in normal and IPF lung tissue, respectively. Results showed that SENP1 expression in CD90^+^ LR-MSCs increased in IPF lung tissue compared with normal tissue (Fig. 2B). These results suggest that SENP1 levels of LR-MSCs increased when LR-MSCs transformed into myofibroblasts in IPF.

### SENP1 upregulated during myofibroblast differentiation of LR-MSC ex vivo

**Fig. 3:**
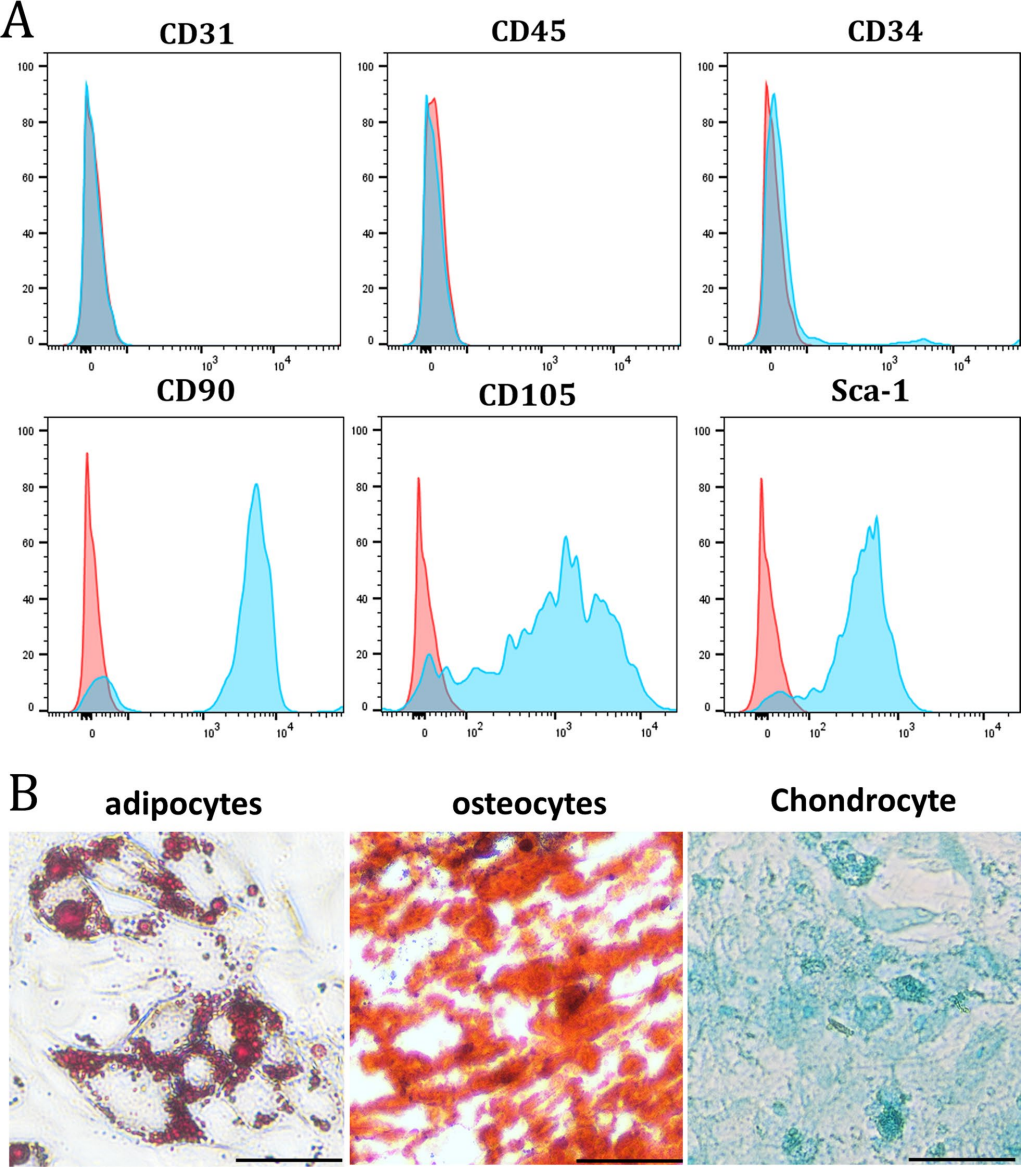
Isolation and identification of mouse LR-MSCs. **A** LR-MSCs isolated from mice lungs were analyzed using flow cytometry. **B** Mouse LR-MSCs were seeded in the appropriate differentiation induction medium. After 21 days of induction for adipocytes, osteocytes, and Chondrocyte differentiation, cells were stained with oil red O, alizarin red and Alcian Blue. Scale bar, 20 μm

In order to detect the myofibroblast differentiation of LR-MSCs and SENP1 level in vitro, we isolated primary mice LR-MSCs for ex vivo experiments. Flow cytometry analysis showed that isolated LR-MSCs expressed surface marker of MSCs Sca-1, CD90, and CD105, but not endothelial marker CD31 or hematopoietic origin marker CD45 (Fig. 3A). These cells can differentiate into adipocytes, osteocytes, and chondrogenic cells (Fig. 3B). Thus, the isolated cells meet the criteria for identifying LR-MSC as described previously.

**Fig. 4:**
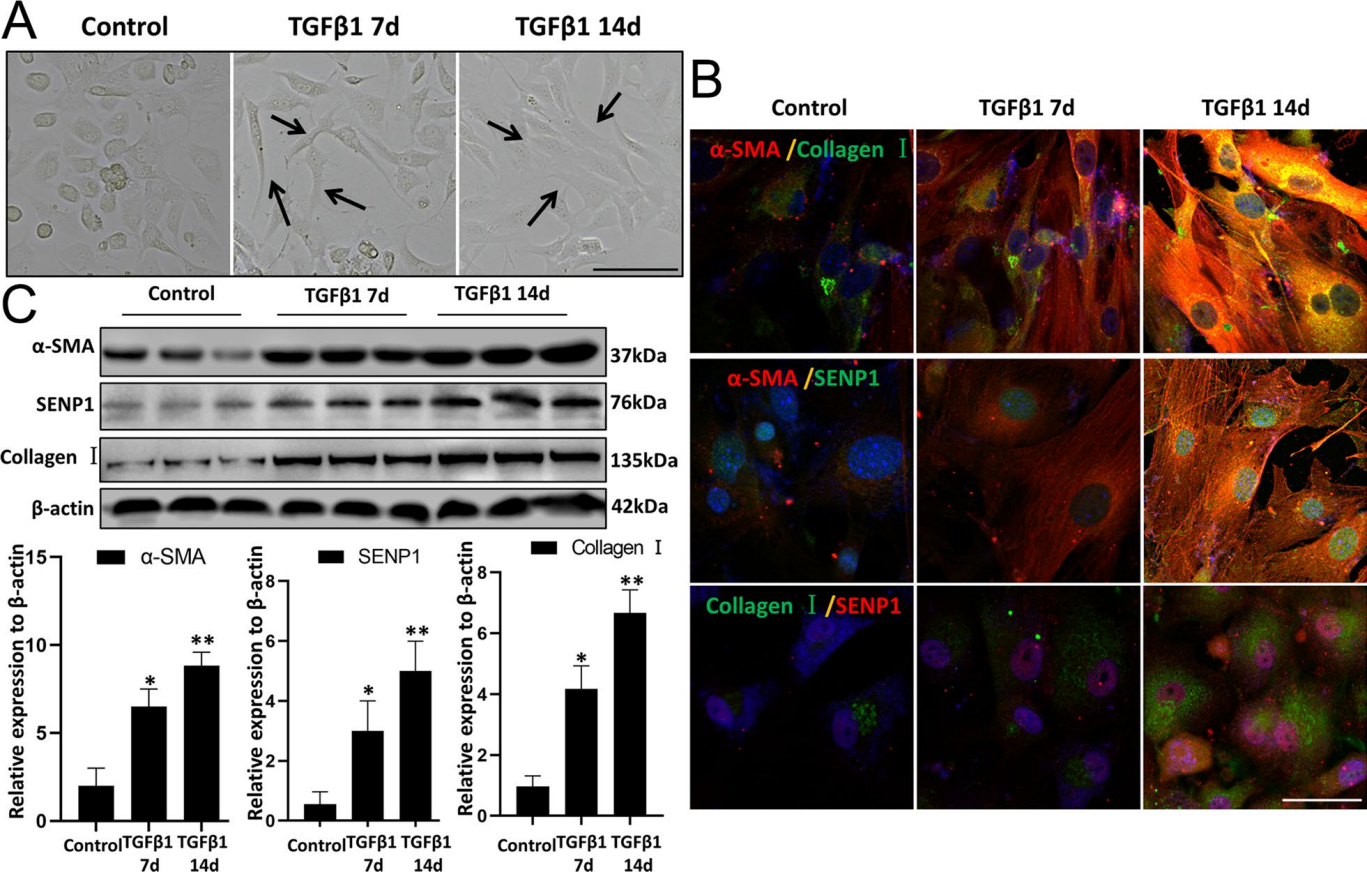
The expression of SENP1 increases in LR-MSCs from mice. **A** LR-MSC morphology was observed using light microscopy after mouse LR-MSCs were treated with TGFβ1 (10 ng/mL) for 7 and 14 d. Black arrows indicate LR-MSCs. **B** Representative images of dual staining for Collagen I (green) and α-SMA (red), SENP1 (green) and α-SMA (red), and dual staining for SENP1 (red) and Collagen I (green). **C** Western blotting was applied to detect the levels of α-SMA, SENP1, Collagen I in LR-MSCs after the TGFβ1 (10 ng/mL) treatment for 14d. Scale bar, 20 μm. *P < 0.05, control group vs. the TGFβ1 7d group; **P < 0.01, control group vs. the TGFβ1 14d group. The results are shown as the means ± SD. Each experiment was performed in triplicate

The classical pro-fibrotic growth factor TGFβ1 was used to induce myofibroblast differentiation of LR-MSCs. After TGFβ1 (10 ng/mL) stimulation for 7 and 14 days, the appearance of LR-MSCs became thicker and longer, which is similar to the morphology of myofibroblasts (Fig. 4A). Immunofluorescence and western blot results also showed that the expression of α-SMA, Collagen I, and SENP1 in LR-MSCs increased in TGFβ1 group compared with that in the control group (Fig. 4B-C).

### Downregulation of *SENP1* inhibit the transformation of LR-MSCs into myofibroblasts

**Fig. 5:**
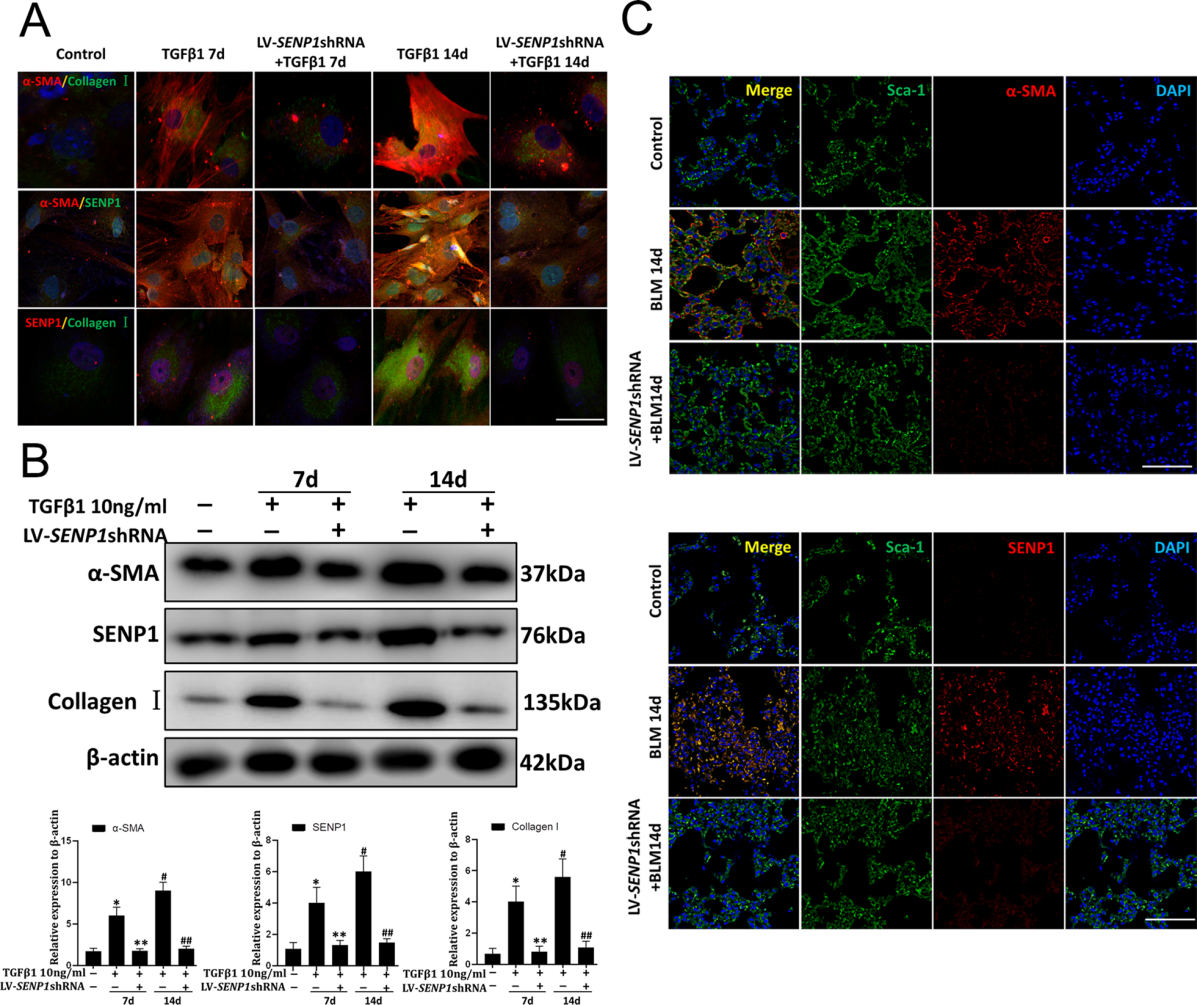
The LR-MSCs-myofibroblast transition was inhibited upon *SENP1* downregulation in vitro and in vivo. **A** Representative images of dual staining in *vitro* for Collagen I (green) and α-SMA (red), SENP1 (green) and α-SMA (red), and dual staining for SENP1 (red) and Collagen I (green). Scale bar, 15 μm. **B** Collagen I, α-SMA, and SENP1 levels were assessed using western blotting analyses. Quantification is shown in the lower panel. **C** Representative images of dual staining in vivo for Sca-1 (green) and α-SMA (red), Sca-1 (green) and SENP1 (red). Scale bar, 50 μm. *P < 0.05, **P < 0.05, #*P* < 0.01, ##*P* < 0.01. *, control group *vs*. the TGFβ1 7d group. #, control group *vs*. the TGFβ1 14d group. **, the *SENP1* knockdown group *vs*. the TGFβ1 7d group. ##, the *SENP1* knockdown group *vs*. the TGFβ1 14d group. Each experiment was performed in triplicate

To determine the effect of SENP1-mediated deSUMOylation on the transdifferentiation of LR-MSCs, LV-SENP1-shRNA was used to downregulate SENP1 expression in mouse LR-MSCs in vitro (Additional file 2: Fig. S1). The immunofluorescence and western blotting results showed that α-SMA, Collagen I, and SENP1 levels were decreased after *SENP1* downregulation under TGFβ1 induction in a time-dependent manner (Fig. 5A–B). In vivo, mice were injected intratracheally LV-SENP1-shRNA to silence *Senp1* expression. RT-PCR showed that this strategy successfully downregulated Senp1 expression (Additional file 3: Fig. S2). Immunofluorescence co-staining results showed that α-SMA levels in Sca-1^+^ LR-MSCs substantially increased at 14 days post BLM induction. Injection intratracheally with the LV-SENP1-shRNA dramatically decreased the levels of α-SMA in Sca-1^+^ LR-MSCs in interstitial lung areas (Fig. 5C).

These results suggested that downregulation of SENP1 could reduce the transformation of LR-MSCs into myofibroblasts.

### Downregulation of SENP1 can restore the reparative effect of LR-MSCs

**Fig. 6:**
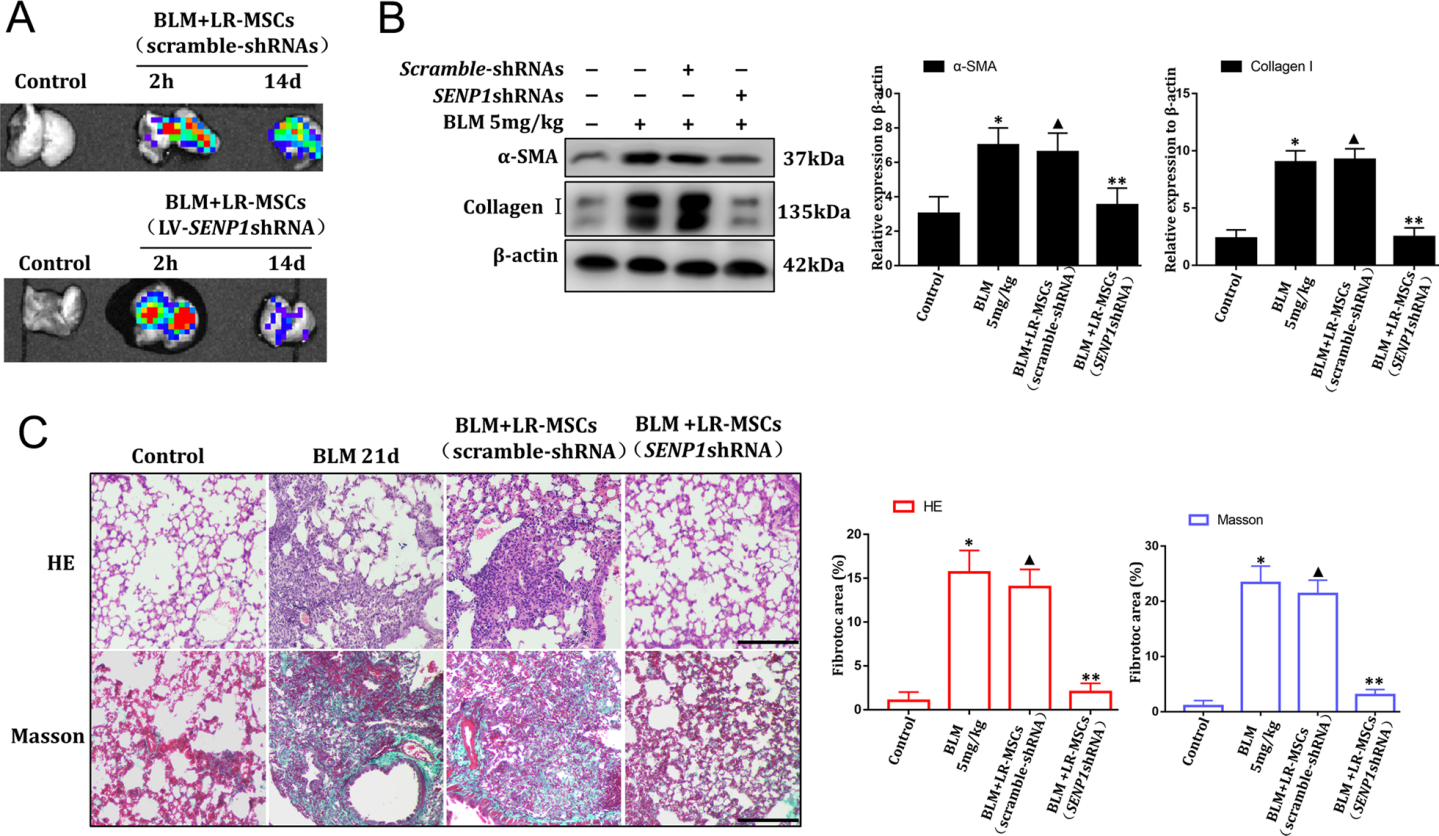
LR-MSCs with *SENP1* downregulation attenuate pulmonary fibrosis. **A** In vivo imaging of GFP + LR-MSCs after transplantation. **B** The expression of α-SMA and Collagen I in mouse lung tissues was analyzed using western blotting in vivo. Quantification is shown in the right panel. **C** The effect of LR-MSCs (LV-SENP1-shRNA) on pulmonary fibrotic lesions was determined using hematoxylin–eosin (H&E) staining and Masson staining. Scale bar, 50 μm. Representative data are shown. Quantification is shown in the right panel. *P < 0.01, ^▲^*P* > 0.05, **P < 0.05. *, the control group *vs*. the BLM group; ^▲^, the LR-MSCs (scramble-shRNAs) group *v*s. the BLM group; **, the LR-MSCs (LV-SENP1-shRNA) group *v*s. the BLM group. The results are shown as the means ± SD

In vivo, BLM modeling, LR-MSCs (scramble-shRNAs), and LR-MSCs (LV-*Senp1*-shRNA) were infused into the bronchus of mice to determine whether downregulation deSUMOylation could improve the healing effects of LR-MSCs. In vivo imaging showed that the labeled LR-MSCs can be traced within 2 h after injection. Most importantly, labeled LR-MSCs could be detected after injection 14d (Fig. 6A). Western blotting results in vivo showed that LR-MSCs could not effectively decrease the levels of α-SMA and Collagen I in the lungs of BLM mice, while LR-MSCs (LV-SENP1-shRNA) downregulated the levels of α-SMA and Collagen I in the lung of BLM mice significantly (Fig. 6B). H&E and Masson staining also showed that compared with the LR-MSCs group, the number of cells and blue-stained collagen fibers in lung interstitium significantly reduced after bronchial instillation of LR-MSCs (LV-SENP1-shRNA) (Fig. 6C).

### SENP1-mediated deSUMOylation is an important target for activation of WNT/β-Catenin and Hedgehog/GLI signaling pathways

**Fig. 7:**
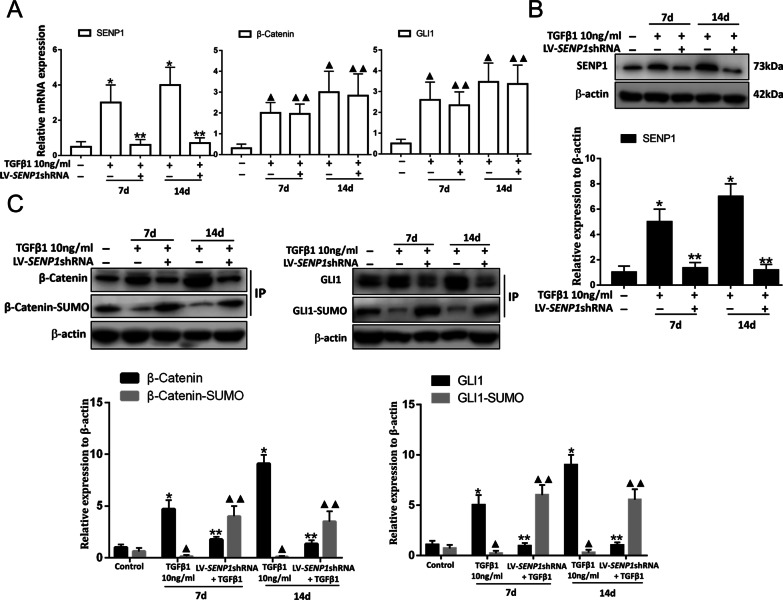
SENP1 regulates LR-MSC myofibroblast transition through both the WNT/β-Catenin and Hedgehog/GLI signaling pathways in vitro. **A** mRNA expression of endogenous *SENP1*, *β-Catenin,* and *GLI1* were assessed using RT-PCR in different groups. RT-PCR analysis indicating that *β-Catenin* and *GLI1* mRNA levels did not change in the presence of LV*-*SENP1-shRNA during TGFβ1 induction. *P < 0.01, **P < 0.05, ^▲^*P* < 0.01, ^▲▲^*P* > 0.05. *, the control group *vs*. the TGFβ1 group; **, the *SENP1* knockdown group vs. the TGFβ1 group; ^▲^, the control group vs. the TGFβ1 group; ^▲▲^, the *SENP1* knockdown group vs. the TGFβ1 group. **B** SENP1 level in total cell lysates was assessed using western blot analyses. *P < 0.01, **P < 0.05. *, the control group vs. the TGFβ1 group; **, the *SENP1* knockdown group vs. the TGFβ1 group. **C** β-Catenin and GLI1 levels in total cell lysates were assessed using western blot analyses. The SUMOylation analysis of the immunoprecipitated β-Catenin and GLI1 proteins. β-Catenin and GLI1 were immunoprecipitated from whole-cell lysates using anti-β-Catenin and anti-GLI1 antibodies, respectively. The blots were probed with SUMO1. *P < 0.01, **P < 0.05, ^▲^*P* < 0.01, ^▲▲^*P* < 0.05. *, the control group *vs*. the TGFβ1 group; **, the *SENP1* knockdown group vs. the TGFβ1 group (β-Catenin and GLI1); ^▲^, the control group *vs*. the TGFβ1 group; ^▲▲^, the *SENP1* knockdown group vs. the TGFβ1 group (β-Catenin-SUMO and GLI1-SUMO). All data are presented as the mean ± SEM of three independent experiments. Quantification is shown in the lower panel. Each experiment was performed in triplicate.

β-Catenin and GLI1 are the key effector proteins of the WNT/β-Catenin and Hedgehog/GLI signaling pathways [11, 12]. We hypothesized that downregulation of *SENP1* might inhibit the levels of β-Catenin and GLI1 through the recovery of SUMOylation. The RT-PCR and western blot results confirmed that LV-SENP1-shRNA efficiently decreased *SENP1* mRNA (Fig. 7A) and protein levels (Fig. 7B) after TGFβ1 induction in LR-MSCs. However, LV-SENP1-shRNA did not affect *β-Catenin* and *GLI1* mRNA levels (Fig. 7A). Moreover, immunoprecipitation results showed that the levels of SUMO1-modified β-Catenin and GLI1 decreased significantly after TGF-β1 induction and increased by *SENP1* knockdown in vitro. The levels of β-Catenin and GLI1 significantly decreased, while the levels of SUMO1-modified β-Catenin and GLI1 were increased in response to *SENP1* knockdown (Fig. 7C). Thus, the inhibition of SENP1-mediated deSUMOylation simultaneously inactivated the WNT/β-Catenin and Hedgehog/GLI pathways.

## Discussion

LR-MSCs have become a hotspot in the field of pulmonary fibrosis research because of their potential for repair and differentiation [8]. The transformation of LR-MSCs into myofibroblasts lose the therapeutic effect of stem cells and induces IPF progression [9, 10]. At present, the specific mechanism regulating the differentiation of LR-MSCs is unclear. It has been reported that activation of several signaling pathways is involved in the transformation of LR-MSCs [11, 12], and SENP1 can upregulate the level of substrate proteins, thereby activating the associated signaling pathway [15, 16]. Therefore, the innovation of this study is the finding that SENP1-mediated deSUMOylation is an important target to regulate the transdifferentiation of LR-MSCs and induce IPF.

The extracellular matrix secreted by myofibroblasts is the direct cause of IPF progression [18]. Previous studies have shown that an important way to use mesenchymal stem cells to treat pulmonary fibrosis is to inhibit the emergence of myofibroblasts [19, 20]. With the development of stem cells in the field of pulmonary fibrosis, the latest research found that LR-MSCs exert their therapeutic function indirectly via repair of lung tissues, but not if they transform into myofibroblasts [9]. In this study, we also observed that LR-MSCs transformed into myofibroblasts in the lung tissue of patients with IPF. This clinical observation suggests that LR-MSCs may play an important role in IPF.

LR-MSCs are located in the lung interstitium, which is distributed around the bronchi and alveoli [21]. The gene expression profile of LR-MSCs is different from that of bone marrow-derived mesenchymal stem cells [22]. LR-MSCs express stemCD90, CD44, and CD105, but do not express CD45 and CD31 in human [21]. These CD90^+^ cells were observed in IPF lung tissue, which was consistent with the characteristics of LR-MSCs rather than stem cells of other origins, such as bronchioalveolar stem cells (BASCs), which cannot transform into myofibroblasts. Currently, there is no commercial mouse LR-MSC cell line. In this study, primary mouse LR-MSCs were isolated successfully using immunomagnetic bead sorting. The results of flow cytometry and stem cell differentiation staining showed that the extracted LR-MSCs were a subtype of mesenchymal stem cells, which was consistent with previous reports.

The activation of multiple signaling pathways can accelerate LR-MSCs transformation into myofibroblasts under specific conditions [23]. Previous studies have shown that most of the key proteins involved in downstream nuclear transcription signaling pathways have SUMO modifications, and the deSUMOylation mediated by SENP1 activates the signaling pathway excessively [24, 25]. Therefore, we speculated that SENP1 might be involved in the transformation of LR-MSCs into myofibroblasts. In the lung tissues of patients with IPF, we found that SENP1 overexpressed during the transformation of LR-MSCs into myofibroblasts. SENP1 was also overexpressed in BLM mice and in the transformation of LR-MSCs induced by TGFβ1. Interestingly, downregulation of *Senp1* expression in LR-MSCs reduced the transformation of LR-MSCs into myofibroblasts. These results suggested that SENP1 is an important target for regulating the transformation of LR-MSCs into myofibroblasts.

Maintaining the normal repair function of MSCs can prevent the appearance of myofibroblasts [26]. Under normal circumstances, like other MSCs, LR-MSCs can maintain lung homeostasis and prevent IPF by repairing damaged lung tissues [9, 27]. In this study, we found that the main reason why LR-MSCs did not perform their repair function was that SENP1 promoted the transformation of LR-MSCs into myofibroblasts. Whether inhibition of SENP1 could restore LR-MSC-mediated repair was also studied. In the present study, bronchial installation of *Senp1*-silenced LR-MSCs could improve the pathology of pulmonary fibrosis in BLM mice. These results suggest that *Senp1*-silenced LR-MSCs could exert the repair function of MSCs and improve IPF pathology.

The transdifferentiation of LR-MSCs is affected by many factors [28]. Under pathological conditions, such as chronic injury, multiple signaling pathways activated by TGF-β1, WNT, tumor necrosis factor-alpha (TNF-alpha), and fibroblast growth factor (FGF) can induce LR-MSCs to transform into myofibroblasts [29–31]. Blocking the activation of a single signal pathway might alleviate pulmonary fibrosis to a certain extent; however, other signal pathways might be overactivated in compensation, resulting in incomplete restoration of the repair function of LR-MSCs. These cytokines can activate WNT/β-Catenin and Hedgehog/GLI signaling pathways and promote the differentiation of LR-MSCs into myofibroblasts [11, 12]. In the above signaling pathways, SUMO modification exists in the downstream signal proteins β-Catenin and GLI1, which promote nuclear transcription [31, 32]. SENP1 can cut the SUMO chain on the lysine residues of β-Catenin and GLI1, thus maintaining the levels of β-Catenin and GLI1 [15, 16]. In this study, we found that downregulation of *Senp1* in LR-MSCs reduced the levels of β-Catenin and GLI1 by restoring their SUMO modification levels. This suggested that SENP1-mediated deSUMOylation might be an important target for regulating the activation of multiple signaling pathways involved in the transdifferentiation of LR-MSCs.

In conclusion, modulating SENP1-mediated deSUMOylation is an effective strategy to control the transdifferentiation direction of LR-MSCs and modify the progress of IPF. However, whether other signaling pathways are essential for the transdifferentiation of LR-MSCs and whether the therapeutic effect of modifying multiple signaling pathways by regulating SENP1 is better than that of single signaling pathway inhibitors needs further clarification.

## Conclusions

Our study explores for the first time the role of SENP1 mediated deSUMOylation in regulating LR-MSCs transdifferentiation through simultaneous effects on multiple related signaling pathways. Interventions targeting SENP1 can restore the repair function of LR-MSCs with potential therapeutic effect on pulmonary fibrosis. However, our study failed to address some relevant questions. First, although our study confirms that LR-MSCs are the source of myofibroblasts, we are unable to state the fraction of this effect that is accounted for at the time of fibrosis. Second, SENP1-mediated deSUMOlyzation is a widespread intracellular mechanism that regulates protein levels. Although we explored the Wnt/β-βcatenin and Hedgehog/GLI pathways, however, the impact of SENP1-mediated deSUMOlyzation on other signaling pathways and in the transformation of LR-MSC to myofibroblasts remain unresolved.

## Supplementary Information

**Additional file 1**. **Table S1**. Characteristics of patients who provided surgical samples.

**Additional file 2**. **Fig. S1**. Senp1 expression assessed using RT-PCR analysis in vitro. *P < 0.01, **P < 0.05. *, control group *vs*. the LV-SENP1-shRNA; **, the TGFβ1 group *vs*. the *SENP1* knockdown group + TGFβ1 group. The results are shown as the means ± SD.

**Additional file 3**. **Fig. S2**. Senp1 expression was assessed using RT-PCR in vivo. *P < 0.01, **P < 0.05. *, the control group *vs.* the LV-SENP1-shRNA group; **, the BLM group *vs.* the LV-*SENP1*-shRNA + BLM group. The results are shown as the means ± SD.

## Data Availability

The datasets used and/or analysed during the current study are available from the corresponding author on reasonable request.

## Abbreviations

IPF: Idiopathic pulmonary fibrosis
LR-MSCs: Lung resident mesenchymal stem cells
SENP1: SUMO specific protein 1
GLI: GLI family zinc finger
SUMOylation: Small ubiquitin-like modifier
