# Supplementary material for: SENP1 regulates the transformation of lung resident mesenchymal stem cells and is associated with idiopathic pulmonary fibrosis progression
Source: Cell Commun Signal. 2022 Jul 14;20:104. doi: 10.1186/s12964-022-00921-4 (PMC9281027; doi:10.1186/s12964-022-00921-4)
Supplement: Supplementary file 2 — Additional file 1. Table S1. Characteristics of patients who provided surgical samples. [file 12964_2022_921_MOESM2_ESM.docx]

**S1**. Characteristics of patients who provided surgical samples

|  | Normal | IPF | *P* |
| --- | --- | --- | --- |
| Number | 6 | 6 | - |
| Gender (M/F) | 4/2 | 5/1 | 0.505^*^ |
| Age (year) | 65.3±13.7 | 73.7±6.5 | 0.205^†^ |
| Body mass index (kg/m^2^) | 26.62±6.8 | 24.26±10.5 | 0.657^†^ |
| Smoker (n) | 4 | 3 | 0.558^*^ |
| Pack-years of smoker | 54.25±9.251 | 49.33±4.041 | 0.436^†^ |
| FEV1, % ref | 93.72±4.653 | 85.07±5.286 | 0.013^†^ |
| FEV1/FVC, % | 91.82±3.604 | 85.11±5.341 | 0.029^†^ |
| DLco, % ref | 92.13±2.021 | 36.70±5.874 | <0.0001^†^ |

*. The chi-square test was used to compare the differences in composition ratio; †. Values are shown as mean ± SD. Comparison of mean value between two groups using two-tailed *t*-test. The statistically significant difference when *p*＜0.05. FEV1, forced expiratory volume in 1 second; FVC, forced vital capacity; DLco, carbon monoxide diffusion capacity.
