# Supplementary figures and images for: SENP1 regulates the transformation of lung resident mesenchymal stem cells and is associated with idiopathic pulmonary fibrosis progression
Source: Cell Commun Signal. 2022 Jul 14;20:104. doi: 10.1186/s12964-022-00921-4 (PMC9281027; doi:10.1186/s12964-022-00921-4)

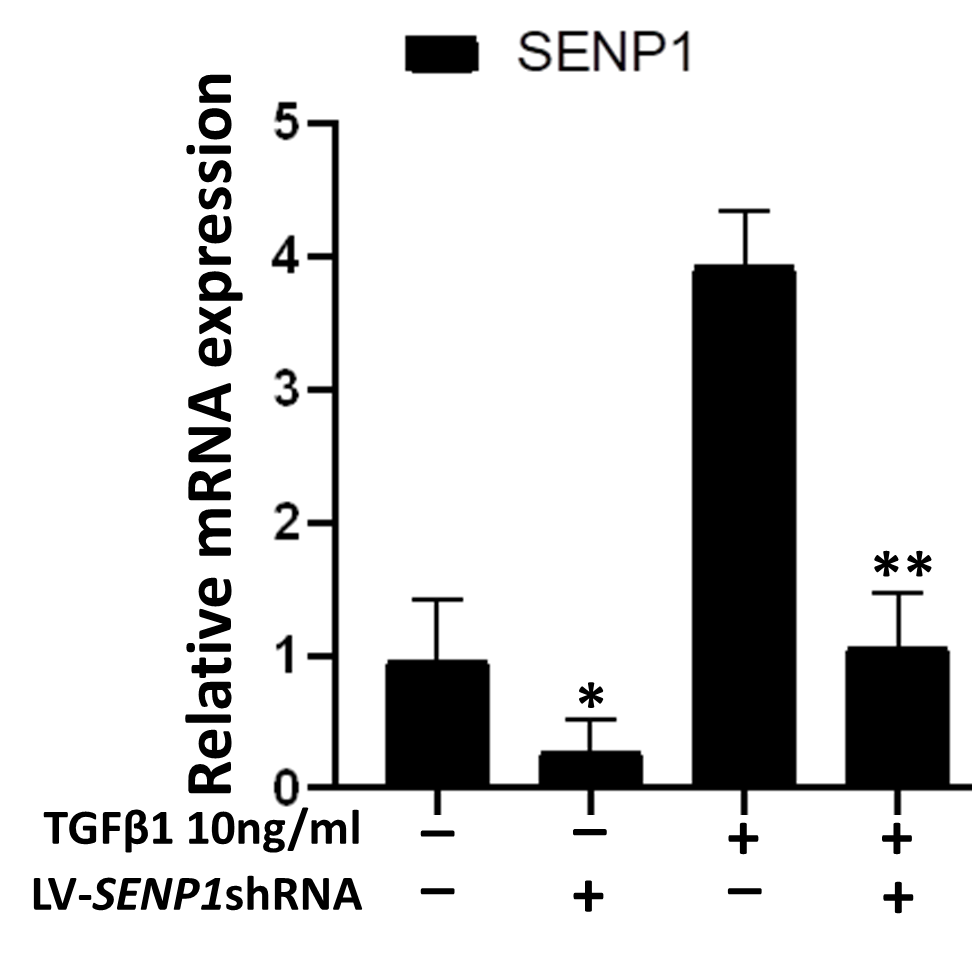

Supplement: Supplementary file 3 — Additional file 2. Fig. S1. Senp1 expression assessed using RT-PCR analysis in vitro. *P < 0.01, **P < 0.05. *, control group vs. the LV-SENP1-shRNA; **, the TGFβ1 group vs. the SENP1 knockdown group + TGFβ1 group. The results are shown as the means ± SD. [file 12964_2022_921_MOESM3_ESM.tif]

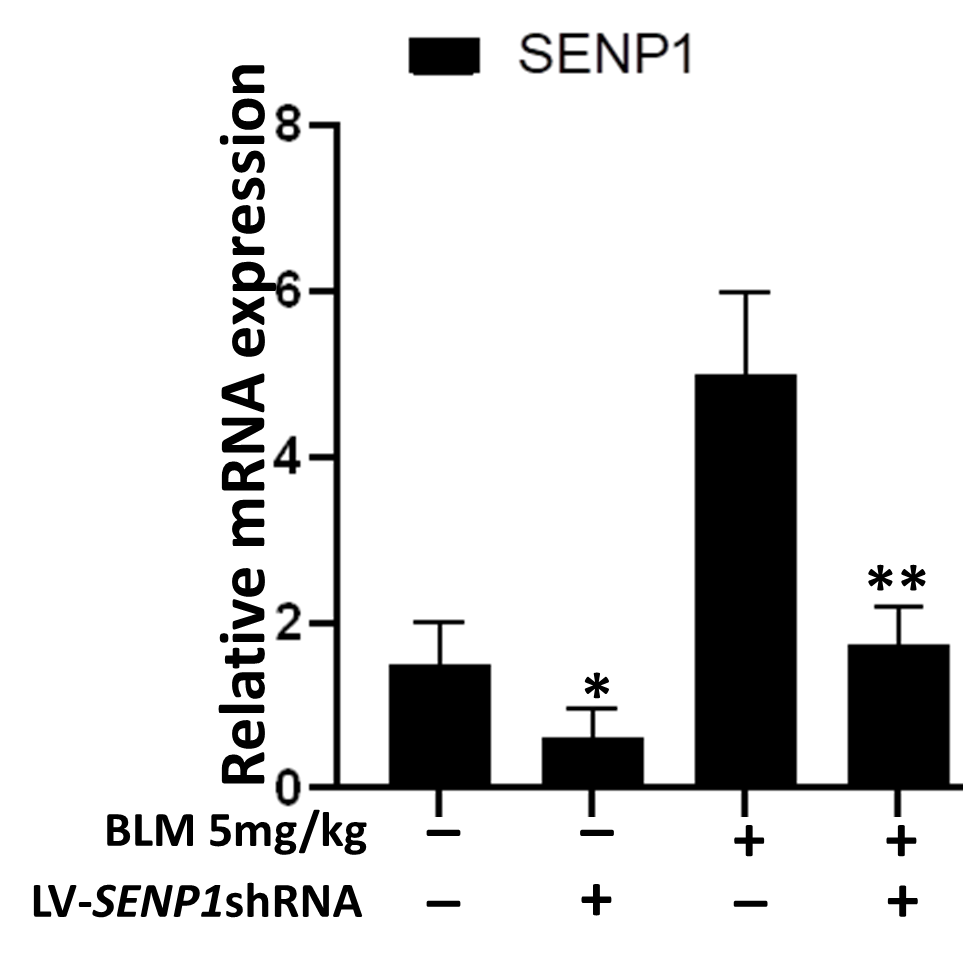

Supplement: Supplementary file 4 — Additional file 3. Fig. S2. Senp1 expression was assessed using RT-PCR in vivo. *P < 0.01, **P < 0.05. *, the control group vs. the LV-SENP1-shRNA group; **, the BLM group vs. the LV-SENP1-shRNA + BLM group. The results are shown as the means ± SD. [file 12964_2022_921_MOESM4_ESM.tif]
